# Functional Interaction Between Toc75 POTRA1 Domain and Tic22‐III in the Intermembrane Space During Chloroplast Protein Import

**DOI:** 10.1002/pld3.70138

**Published:** 2026-02-12

**Authors:** Rajneesh Singhal, Danny J. Schnell

**Affiliations:** ^1^ Department of Plant Biology Michigan State University East Lansing Michigan USA

**Keywords:** chaperones, chloroplasts, POTRA, protein import

## Abstract

Chaperones are essential for facilitating the import of nuclear‐encoded precursor proteins into chloroplasts. In the intermembrane space (IMS) of the chloroplasts, this process is mediated by the transport‐associated domains (POTRA) of the translocon at the outer envelope membrane (Toc75) and translocon at the inner envelope membrane (Tic22) proteins. The present work aims to understand the interaction between the Toc75 POTRA domain and Tic22 in the IMS and determine their relationship in facilitating protein import. Expression of the POTRA1 domain deleted *TOC75* (*TOC75ΔP1*) in the *tic22‐III* mutant background resulted in a more severe phenotype than the individual mutants, indicating that the two proteins functionally interact in the IMS. Using an insulin aggregation assay, we have demonstrated that Tic22‐III also possesses chaperone‐like activity. In vitro import experiments suggest that *TOC75ΔP1/tic22‐III* plants are compromised in importing stromal and thylakoid membrane proteins. Therefore, we propose that the Toc75 POTRA domains and Tic22‐III both provide chaperone activity necessary to prevent the misfolding of incoming pre‐proteins, acting as chaperones and facilitating the protein import process through the IMS of the chloroplast.

**Abbreviations**
IMS: intermembrane space
TIC: translocon at the inner envelope membrane
TOC: translocon at the outer envelope membrane

## 1 Introduction

Chloroplast biogenesis and function are reliant on the delivery of 2000–3000 nuclear‐encoded proteins, which are synthesized in the cytosol with N‐terminal chloroplast transit peptide (cTP) as pre‐proteins and translocated across the envelope membranes by the two membrane translocons: translocon at the outer envelope of the chloroplasts (TOC) and translocon at the inner envelope membrane of the chloroplasts (TIC) (Ling et al. 2015; Leister 2016; Richardson et al. 2017; Nakai 2018; Richardson and Schnell 2020; Sun and Jarvis 2023; Xing et al. 2025). The TOC complex includes GTPase‐activity‐possessing receptor components, Toc34 and Toc159, which recognize transit peptide and direct the pre‐protein to Toc75, a β‐barrel membrane protein forming the import channel (Perry and Keegstra 1994; Schnell et al. 1994; Schleiff et al. 2002; Oreb et al. 2011; Day et al. 2014; Schnell 2019). The TIC machinery is less well defined, as two different complexes have been proposed to form the TIC complex. The first complex consists of Tic110, Tic40, and Tic20 as core components, where Tic110 functions as a scaffold for docking the stromal chaperones (Kessler and Blobel 1996; Ma et al. 1996; Inaba et al. 2003; Chou et al. 2006). The second complex consists of Tic12, Tic20, Tic35, Tic214, Tic100, and Tic56, where Tic20 forms the import channel at the inner envelope membrane (IEM) (Kikuchi et al. 2013; Liang, Jin, et al. 2024; Xing et al. 2025). In addition, a recently discovered protein at the IEM, Tic236, links the TOC and TIC complexes by interacting with the POTRA domains of the Toc75 protein and Tic110 (Chen et al. 2018).

Chaperones are required to maintain proteins in an unfolded import‐competent conformation during transit across biological membranes in chloroplasts. In the cytosol, chaperones like heat shock protein 70 (Hsp70) and Hsp90 assist in targeting pre‐proteins to the chloroplast outer membrane. In the stroma, chaperone activity is provided by two independent motor complexes. One complex is formed by chaperones such as chloroplast Hsp70, chloroplast Hsp90C, and Hsp93/Caseinolytic protease C (ClpC) (Constan et al. 2004; Inaba et al. 2005; Kovacheva et al. 2007; Inoue et al. 2013; Flores‐Pérez et al. 2016; Huang et al. 2016; Li et al. 2020), and the other complex is formed by Ycf2/FtsHi AAA+ ATPase motor module (Kikuchi et al. 2018; Liang, Jin, et al. 2024; Liang, Zhan, et al. 2024).

However, the chaperone complex facilitating uptake and translocation across the IEM is less well‐defined. Several proteins, including Tic22, Toc64, Tic100, Tic236, Toc12, iHsp70, and Toc75, have been implicated in forming a complex in the intermembrane space (IMS). One of the proposed complexes consists of Toc64, Toc12, Tic22, and iHsp70 proteins (Becker et al. 2004; Qbadou et al. 2007). However, later studies have shown that both Toc12 and Hsp70 are located in the chloroplast stroma and therefore cannot form a complex at the IMS (Ratnayake et al. 2008; Su and Li 2008, 2010; Chiu et al. 2010). The other proposed complex in the IMS consists of the POTRA domains of Toc75, Tic22, and Tic236 proteins (Paila et al. 2016; Chen et al. 2018; Sun and Jarvis 2023). Tic236 is a 230‐kDa protein that is integrated into the inner envelope membrane. It projects its C‐terminal DUF90 into the IMS, which binds to Toc75, thus acting as a bridge connecting the TOC and TIC complex components (Chen et al. 2018). Toc75 has three POTRA domains. Toc75 interacts with pre‐proteins at all stages of import, and its POTRA domains have been shown to possess chaperone activity, which helps maintain the pre‐proteins in an unfolded state (O'Neil et al. 2017). Notably, POTRA2–3 domains exhibit chaperone activity comparable to all three POTRA domains (POTRA1–3). Tic22, found in plastids across plants, algae, and Apicomplexa parasites, is essential for survival in apicomplexans and cyanobacteria, exhibiting holdase activity and involvement in the biogenesis of the outer envelope membrane (Glaser et al. 2012; Tripp et al. 2012; Brouwer et al. 2019). The POTRA domains of Toc75 have been shown to physically interact with Tic22‐III and the precursor of the small subunit of rubisco in in vitro studies (Paila et al. 2016).

Since both POTRA domains of Toc75 and Tic22‐III are located in the IMS of the chloroplasts and interact, we hypothesized that the two proteins function as a complex to facilitate the transit of preproteins from TOC to TIC across the IMS. In this work, we demonstrate that Tic22‐III of Arabidopsis exhibits chaperone activity. Through genetic and biochemical analyses of *tic22‐III/TOC75ΔP1* double mutant plants, we provide evidence that Toc75 POTRA domains and Tic22‐III both provide chaperone activity, which is crucial for efficient chloroplast protein import and overall chloroplast development.

## 2 Materials and Methods

### 2.1 Plant Growth and Generation of Double Mutant Plants

The Arabidopsis seeds were surface‐sterilized, stratified for 48 h at 4°C, and grown on 0.8% Phyto agar plates containing 0.5× Murashige and Skoog (MS) media with 1% sucrose in a growth chamber at 22°C and a 16‐/8‐h light/dark cycle. The *T‐DNA* insertion lines *attic22‐III (GK387‐C03)* and *attic22‐IV (GK810‐F06)* were obtained from GABI‐KAT (Rosso et al. 2003) and have been previously characterized as at*tic22‐III‐I* and at*tic22‐IV‐2*, respectively (Kasmati et al. 2013; Rudolf et al. 2013). The POTRA 1 deletion plants at*TOC75ΔP1* have been described previously (Paila et al. 2016). The at*tic22‐III*/at*TOC75ΔP1* and at*tic22‐IV*/at*TOC75ΔP1* plants were generated by crossing at*tic22‐III* or at*tic22‐IV* plants with at*TOC75ΔP1* hemizygous plants. F3 plants homozygous for at*tic22‐III*/at*TOC75ΔP1* or at*tic22‐IV*/at*TOC75ΔP1* in atTOC75 WT background were selected based on segregation analysis.

### 2.2 Chloroplast Isolation and Protein Import

Intact chloroplasts for protein import analysis were isolated from 13‐ to 14‐day‐old seedlings as described previously (Aronsson and Jarvis 2002; Wang et al. 2008). The radiolabeled substrates (preSSU and preE1α) for the import reaction were generated using a coupled transcription/translation rabbit reticulocyte lysate system containing ^35^S‐methionine according to the manufacturer's recommendation (Promega, Madison, WI). Import reactions were carried out as described previously (Aronsson and Jarvis 2002). Briefly, the import reaction was carried out at 26°C in an Eppendorf tube containing 25‐μg chloroplasts, 2‐mM ATP, 10‐mM methionine, 1‐mM DTT, and import buffer (330‐mM sorbitol, 50‐mM HEPES‐KOH, pH 7.5, 25‐mM KOAc, and 5‐mM MgAOc) in a total volume of 100 μL. The import reaction was initiated by adding 2 μL of in vitro‐translated radiolabeled precursor proteins, and the reaction was carried out for 2, 5, or 10 min. Afterward, the reaction was stopped by adding an excess of cold import buffer. The chloroplasts were washed three times in import buffer, then mixed with 2× SDS‐PAGE sample buffer, and resolved by SDS‐PAGE. The samples were analyzed by phosphor imaging.

### 2.3 Protease Treatments

Thermolysin treatments of intact chloroplasts were performed as described previously (Flores‐Pérez and Jarvis 2017). Following the import reaction, the chloroplast pellet was resuspended in 100 μL of ice‐cold HS buffer (50‐mM HEPES‐KOH, 330‐mM sorbitol, pH 7.5). Thermolysin was added at a concentration of 100 μg/mL and incubated for 30 min on ice. The reaction was quenched using 0.5‐M EDTA to a final concentration of 20 mM, and the chloroplasts were reisolated on a 40% Percoll cushion and washed with ice‐cold HS buffer containing EDTA.

### 2.4 Immunoblot Analysis

Immunoblot analysis was carried out using the same chloroplasts used for protein import analysis. Total protein (10 or 20 μg) was resuspended in 2× SDS‐PAGE sample buffer and resolved on SDS‐PAGE. The proteins were transferred to PVDF‐immobilon membranes. Immunoblots were imaged using LICOR Odyssey CLx imaging system and quantified using ImageJ. Antisera to Lhc's were from Agrisera. Licor 800CW anti‐rabbit secondary antibodies were used at 1:15,000 dilution.

### 2.5 Chlorophyll Measurement

Total chlorophyll was extracted by adding pre‐weighed leaves to a 1.5‐mL tube containing 1 mL of 80% acetone solution. The seedlings were crushed using micro‐pestles and incubated in the dark for 24 h followed by centrifugation at 3000 rpm for 10 min. The supernatant was collected and the chlorophyll absorption was measured at an excitation of 645 and 663 nm using a spectrophotometer. The chlorophyll concentration was calculated according to Arnon's equation, where V = volume of extract (mL) and W = weight of seedlings (g) (Arnon 1949).

$$\mathrm{Ca}+b\;\left(\mathrm{mg}/g\right)=\left[8.02\times A663+20.20\times A645\right]\times V/1000\times W\;\left(\text{Chlorophyll}\;a+b\right)$$

### 2.6 Expression and Purification of Tic22 Protein

The mature fragment of Tic22‐III was expressed from pET21a vectors in BL21(DE3) cells with a c‐terminal hexahistidine tag. The expressed proteins were purified from inclusion bodies as described in (O'Neil et al. 2017) with some modifications. Briefly, an aliquot of overnight‐ grown starter culture was added to 1 L of LB + 100 μg/mL ampicillin and was allowed to grow till OD_600_ of the culture reached 0.6–0.8, at which point protein expression was induced by adding 1 mM isopropyl β‐D‐1‐thiogalactopyranoside at 37°C. After 3 h of further growth, the cells were harvested by centrifugation at 8000 × *g* for 15 min, and the pellet was resuspended in 1/10 volume of TGE buffer (25‐mM Tris–HCl, pH 8.0, 10‐mM EDTA, 50‐mM glucose) supplemented with 200 μg/mL final concentration of lysozyme. The cells were incubated at room temperature for 20 min, followed by homogenization with a probe sonicator. The lysed suspension was centrifuged at 14,500 × *g* for 20 min, and the inclusion bodies pellet was washed three times with TGE + 1% triton‐100 buffer, and once without detergent, and finally resuspended in binding buffer (50‐mM sodium phosphate pH 7.5, 100‐mM NaCl) having 6‐M urea. Since the inclusion bodies pellet was mostly clean with Tic22‐III protein, it was directly dialyzed three times against binding buffer at 4°C. The Tic22‐III protein was then quantified and concentrated using an Ultracel centrifugal filter with a 10‐kDa cutoff for use with the insulin aggregation assay.

### 2.7 Insulin Aggregation Assay

The insulin aggregation assay was carried out as described previously (Glaser et al. 2012). Briefly, 35 μL of insulin was mixed with Tic22‐III in either a 1:0.25 or 1:1 ratio of insulin:Tic22 in PBS buffer in a total volume of 100 μL in a 96‐well plate. The addition of 20‐mM DTT initiated the reaction, and the aggregation of insulin B was monitored over time by measuring the absorbance at 360 nm with a Spectramax microplate reader (Molecular Devices) at 25°C while shaking. The light scattering of insulin in the presence of Tic22 was normalized to that of insulin and 20‐mM DTT in the absence of Tic22. Experiments were performed in triplicate, and the data were averaged and plotted using Microsoft Excel.

### 2.8 Statistical Analysis

All experiments were performed in triplicate and the data were analyzed by calculating averages and standard error from replicates in Excel. For each figure, the differences between genotypes were evaluated using one way analysis of variance (ANOVA) followed by Tukey's honestly significant difference (HSD) multiple comparison test implemented in estimated marginal means (*emmeans*) package in R (version 4.5.0) (Lenth et al. 2025). Compact letter displays were used to indicate statistically significant groupings (α = 0.05).

## 3 Results

### 3.1 Tic22‐III Possesses Chaperone‐Like Activity

**FIGURE 1:**
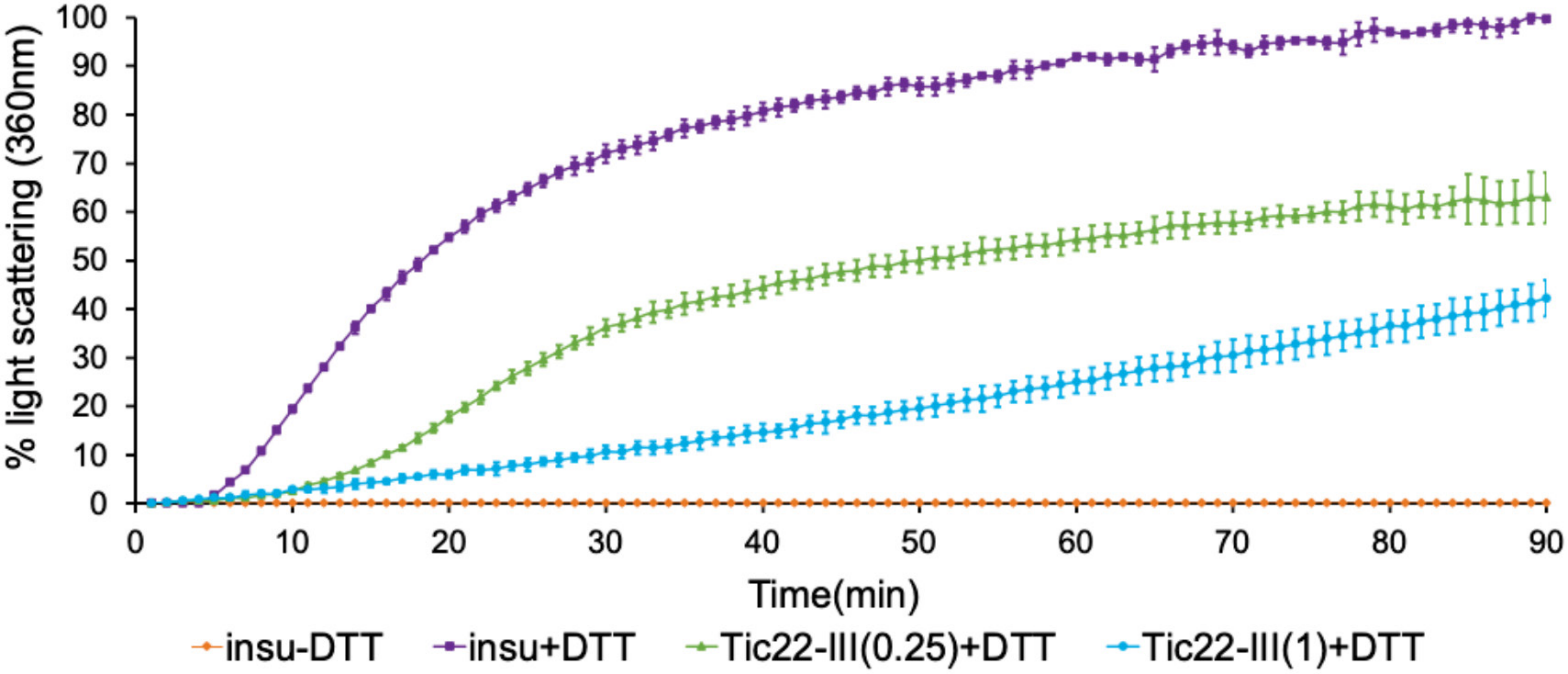
Tic22‐III possesses chaperone‐like activity. Insulin (50 μM) was incubated with Tic22‐III at the indicated Tic22:insulin ratio of 0.25:1 or 1:1 for 90 min. Light scattering (absorbance at 360 nm) due to aggregation of insulin B was measured by adding 20‐mM DTT to the reaction. Light scattering observed at 360 nm due to insulin B aggregation in the presence of DTT alone was set to 100%. As a negative control, light scattering was also monitored for Insulin B in the absence of DTT. Data are the means ± SD (*n* = 3).

Previous research from various groups has shown that Tic22 belongs to a category of small chaperones and functions as a holdase (Glaser et al. 2012; Brouwer et al. 2019). To investigate whether Tic22 from higher plants has chaperone‐like activity, both Tic22‐III and Tic22‐IV were produced in bacteria and purified. Tic22‐IV could not be maintained in soluble form under conditions suitable for the assay; therefore, we focused on Tic22‐III for further work. To determine if Tic22‐III possesses chaperone‐like activity, we performed an in vitro chaperone assay using insulin as a model substrate (Glaser et al. 2012; O'Neil et al. 2017). Insulin is a dimer protein with two peptide chains, A and B, linked by two disulfide bonds. The addition of 20‐mM DTT to insulin reduces the disulfide bonds between the A and B chains, leading to aggregation of the insulin B chain. This aggregation leads to an increase in light scattering, which is monitored over time at 360 nm. The chaperone activity of Tic22 was assessed by its ability to prevent aggregation of insulin B‐chain. When insulin was incubated with Tic22‐III at a molar ratio of 1:0.25, a 40% reduction in aggregation was observed. On further increasing the ratio of insulin to Tic22‐III to 1:1, a 60% reduction in aggregation was observed (Figure 1). In similar assays, purified Toc75 POTRA1 recombinant protein showed no chaperone activity (O'Neil et al. 2017). These results suggest that Tic22‐III, like the POTRA domains of Toc75, possesses chaperone‐like activity and could function in the IMS of chloroplasts to prevent the misfolding of incoming precursor proteins.

### 3.2 The Phenotype of *tic22‐III × TOC75ΔP1* Plants Suggests a Functional Interaction Between Tic22 and Toc75

**FIGURE 2:**
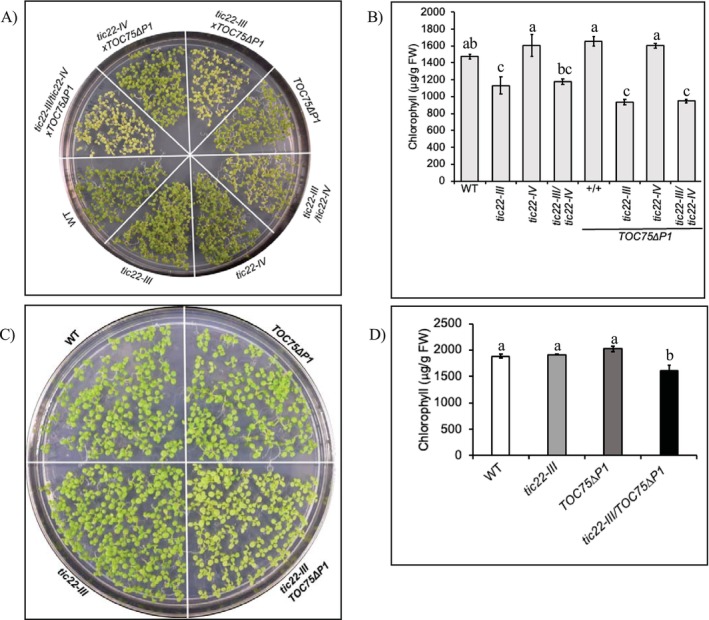
Phenotypic analysis of WT, *tic22‐III*, *tic22‐IV*, *tic22‐III/tic22‐IV*, *TOC75*Δ*P1*, *tic22‐III/TOC75ΔP1*, *tic22‐IV/TOC75ΔP1*, and *tic22‐III/tic22‐IV/TOC75ΔP1* plants in a TOC75 WT background. (A) Phenotypes of 7‐day‐old seedlings grown on MS plates. (B) Total chlorophyll levels in 7‐day‐old seedlings. (C) Phenotypic analysis of 13‐day‐old seedlings. (D) Total chlorophyll levels in 13‐day‐old seedlings. Different letters indicate statistically significant differences among genotypes as determined by one‐way ANOVA followed by Tukey's HSD post hoc test (α = 0.05).

Since the Tic22‐III and POTRA domains of Toc75 possess chaperone‐like activity, and the two proteins interact physically (Paila et al. 2016), we hypothesized that they form a functional complex in the IMS of chloroplasts. We first investigated the genetic interactions between Toc75 and Tic22 proteins. The *tic22‐IV* mutants cannot be distinguished from the wild type (*Col‐0*), but the *tic22‐III‐2* mutants have a pale phenotype during early development (Kasmati et al. 2013; Rudolf et al. 2013). For clarity, these lines will be referred to as *tic22‐IV* and *tic22‐III*. The double mutant *tic22‐III/tic22‐IV* is smaller in size than control plants and shows a modest protein import defect (Kasmati et al. 2013; Rudolf et al. 2013). The three Toc75 POTRA domains are essential for plant survival since deletion of a single POTRA domain cannot complement the lethal *toc75* null mutant. Plants expressing a protein where the first POTRA domain (POTRA 1) is deleted, *TOC75ΔP1*, in a *toc75* heterozygous background exhibit significant dominant‐negative phenotypes, as evidenced by their pale appearance and reduced growth rates (Paila et al. 2016). To investigate the interaction between *tic22* and *toc75* defects, we crossed plants expressing *TOC75ΔP1* in a WT background with *tic22‐III* and *tic22‐IV* plants and examined the F3 generation in the homozygous *tic22* background. We grew all possible parental and mutant/transgenic combinations on MS plates for 7 days to compare their phenotypes (Figure 2A,B). *tic22‐IV* mutants and *TOC75ΔP1* seedlings are indistinguishable from WT, while *tic22‐III* mutants show a pale phenotype and a deficiency in chlorophyll (Figure 2A,B). However, while *tic22‐IV/TOC75ΔP1* seedlings are phenotypically similar to *TOC75ΔP1*, the *tic22‐III/TOC75ΔP1* plants have an enhanced chlorotic phenotype similar to *tic22‐III*. Interestingly, while *tic22‐III* seedlings recovered from chlorophyll defect by day 13, the *tic22‐III/TOC75ΔP1* seedlings fail to recover and retain significantly lower chlorophyll content than all other genotypes (Figure 2C,D). The triple mutant *tic22‐III/tic22‐IV/TOC75ΔP1* plants are indistinguishable from *tic22‐III/TOC75ΔP1* plants. The failure of *tic22‐III/TOC75ΔP1* seedlings to recover from the chlorophyll defect implies a specific functional interaction between Tic22‐III and the POTRA1 domain of Toc75 (Figure 2D).

### 3.3 Chloroplasts From *tic22‐III/TOC75ΔP1* Plants Show Reduced Protein Import Capacity

**FIGURE 3:**
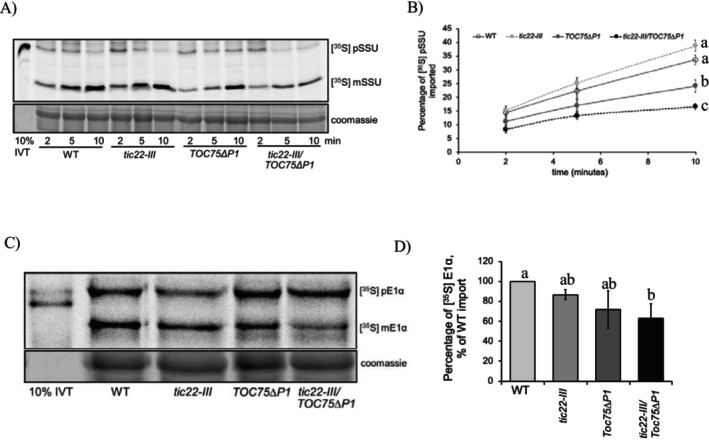
Import of [^35^S] radiolabeled substrates with chloroplasts isolated from WT, *tic22‐III, TOC75ΔP1*, and *tic22‐III/TOC75ΔP1* plants. (A) Time course of the import of in vitro‐translated [^35^S] pre‐pSSU. In the top panel, the chloroplast proteins were separated on an SDS gel and analyzed by phosphor imaging. Ten percent IVT represents 10% of the [^35^S] pSSU added to each reaction. (B) Quantification of [^35^S] pre‐pSSU import reactions. The data points represent the mean of three experiments; error bars represent standard error. (C) Import of [^35^S] preE1α at 10 min. (D) Quantification of [^35^S] preE1αimport reactions. The data points represent the mean of three experiments, and the error bar represents the standard error. Different letters indicate statistically significant differences among genotypes as determined by one‐way ANOVA followed by Tukey's HSD post hoc test (α = 0.05). For (B), the significance test was carried out for 10‐min time point.

Previous studies have demonstrated that the deletion of POTRA domains in the Toc75 protein reduces the rate of precursor protein import. However, the *tic22‐III* single mutants did not show reduced import of precursor proteins into the chloroplasts (Kasmati et al. 2013; Rudolf et al. 2013; Paila et al. 2016). We performed in vitro protein import assays to understand how import is affected in *tic22‐III/TOC75ΔP1* plants. Intact chloroplasts were isolated from WT, *tic22‐III*, *TOC75ΔP1*, and *tic22‐III/TOC75ΔP1* plants and incubated with a radiolabeled precursor protein pSSU (precursor of the small subunit of Rubisco). The import reactions were carried out for 2, 5, and 10 min, and the amount of mature protein imported into the chloroplasts was quantified. Analysis of the results revealed that the import of pSSU in *tic22‐III* mutant plants is similar to that in wild‐type (WT) plants (Figure 3A,B). However, the import of pSSU was reduced by ~28% in *TOC75ΔP1* and ~50% in *tic22‐III*/*TOC75ΔP1* plants compared to WT after 10 min of import. The rate of import is slower in *tic22‐III/TOC75ΔP1* plants compared to *TOC75ΔP1* plants, indicating that the loss of *tic22* enhances the import defect associated with *TOC75ΔP1*. We also examined the import of a precursor of the E1α subunit of pyruvate dehydrogenase ([^35^S] preE1α), an enzyme in chloroplasts. In this case, we also observed a significantly reduced import capacity in *tic22‐III/TOC75ΔP1* plants (Figure 3C,D).

### 3.4 Tic22‐III and Toc75 Are Important for the Import of Hydrophobic Proteins Into Chloroplasts

**FIGURE 4:**
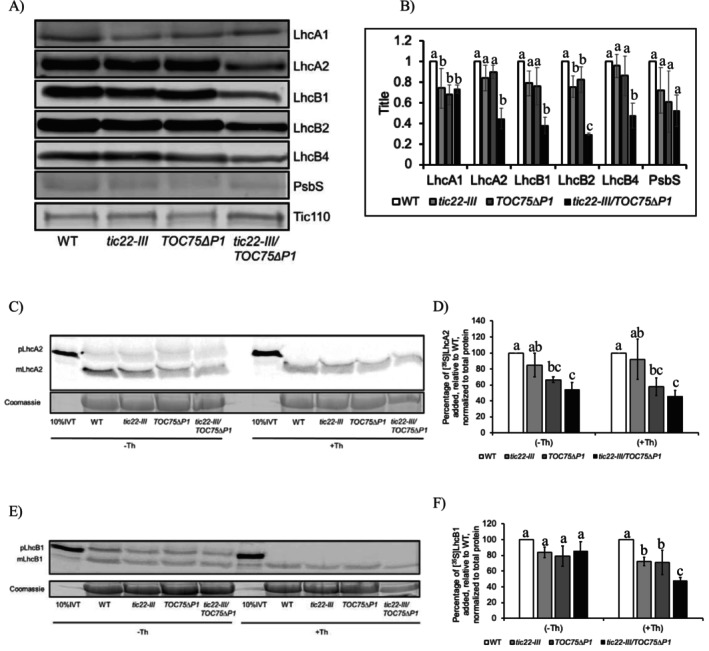
Immunoblot analysis and import of [^35^S] radiolabeled substrates with chloroplasts isolated from 9‐day‐old WT, *tic22‐III*, *TOC75ΔP1*, and *tic22‐III/TOC75ΔP1* plants. (A) 50 μg of total proteins from 7‐day‐old WT, *tic22‐III*, *TOC75ΔP1*, and *tic22‐III/TOC75ΔP1* plants were immunoblotted using antisera indicated at the left. (B) Relative quantification of protein accumulation. The images were captured using a LICOR‐image Odyssey and quantified with the inbuilt software. The samples were normalized to Tic110 in each sample and plotted as a fold change relative to the wild type (WT). (C) Import of in vitro translated [^35^S] pre‐LhcA2. The chloroplasts were separated on SDS gel and analyzed by phosphor imaging. Coomassie‐stained samples indicate equal loading. Ten percent IVT represents 10% of the [^35^S] pLhcA2 added to each reaction. (D) Quantification of import of [^35^S] pLhcA2 in vitro translated reactions normalized to total protein and represented as relative to WT. (E) Import of [^35^S] pre‐LhcB1. The chloroplasts were separated on SDS gel and analyzed by phosphor imaging. Coomassie‐stained samples indicate equal loading. Ten percent IVT represents 10% of the [^35^S] pLhcB1 added to each reaction. (F) Quantification of in vitro translated [^35^S] pre‐LhcB1 reactions normalized to total protein and represented as relative to WT. Quantification of (B), (D), and (F) represents the mean of three experiments; error bars represent the standard deviation. (−Th) and (+Th) show without and with thermolysin treatment. Different letters indicate statistically significant differences among genotypes as determined by one‐way ANOVA followed by Tukey's HSD post hoc test (α = 0.05).

Since Tic22‐III and POTRA domains of Toc75 possess chaperone‐like activity, we hypothesized that a functional interaction between the two in the IMS would facilitate the import of hydrophobic proteins into the stroma. To investigate this, we performed immunoblot analysis on components of Light‐harvesting chlorophyll‐binding proteins (LhcA's and LhcB's) along with PsbS proteins involved in the assembly of Photosystem I and Photosystem II, respectively, which are among the hydrophobic proteins in the chloroplasts. The immunoblot analysis revealed a significant reduction (30%–70%) in the accumulation of LhcA1, LhcA2, LhcB1, LhcB2, and LhcB4 proteins in the *tic22‐III/TOC75∆P1* plants compared to the wild type, while we did not observe any significant reduction in PsbS protein (Figure 4A,B). Interestingly, the reduction in the accumulation of LhcA2, LhcB1, LhcB2, and LhcB4 in *tic22‐III/TOC75∆P1* plants compared to *tic22‐III* or *TOC75∆P1* plants ranged from 50% to 70%, suggesting that the combined chaperone activity conferred by these two proteins at the inner envelope membrane of the chloroplasts is important for the import of hydrophobic proteins (Figure 4A,B). Altogether, the immunoblot analysis indicates that there is perturbation in the accumulation of hydrophobic proteins such as Lhc's.

Since the *tic22‐III/TOC75∆P1* plants showed reduced accumulation of photosystem I and II components in immunoblot analysis, we performed an in vitro protein import assay to investigate whether this is due to reduced efficiency in importing the substrate proteins. We used [^35^S] methionine radiolabeled LhcA2 and LhcB1 as substrate and intact chloroplasts from 9‐day old WT, *tic22‐III*, *TOC75ΔP1*, and *tic22‐III*/*TOC75ΔP1* plants. The import was carried out for 20 min, and the reaction was then divided into two halves. In the first half, thermolysin was added to degrade any preprotein that had not yet been imported into the chloroplasts. In the second half, an equivalent amount of buffer was added. As shown in Figure 4C–F, there was a significant decrease in the import rate of both LhcA2 (Figure 4C,D) and LhcB1(Figure 4E,F) in *tic22‐III*/*TOC75ΔP1* plants compared to WT. However, the extent of the reduction compared to *tic22‐III* or *TOC75ΔP1* plants differed between the two proteins. For LhcA2, the rate of import in *tic22‐III*/*TOC75ΔP1* plants was comparable to that of *TOC75ΔP1* mutants but lower than *tic22‐III*. In contrast, LhcB1 import in *tic22‐III*/*TOC75ΔP1* plants was significantly reduced compared to both *tic22‐III* or *TOC75ΔP1* single mutant plants (+Th). This defect in import suggests that both Tic22‐III and POTRA1 domains of Toc75 protein cooperate in the IMS for the import of hydrophobic proteins.

Altogether, these data demonstrate that Tic22‐III and Toc75 function in a cooperative manner for the import of photosynthetic and non‐photosynthetic substrates, and defects in both proteins lead to more severe impairments in chloroplast development.

## 4 Discussion

Small chaperones play a crucial role in protein translocation across biological membranes, preventing misfolding in the intermembrane space (IMS) of various cellular compartments, such as the bacterial periplasm (e.g., SurA, Skp, BamB) and the mitochondrial IMS (e.g., Tim9, Tim10) (Merdanovic et al. 2011; Weinhäupl et al. 2018; Kim et al. 2021). In the IMS of the chloroplasts, the POTRA domains of Toc75 have been shown to have chaperone activity (O'Neil et al. 2017). Additionally, Tic22 from apicomplexan plastids and cyanobacteria also functions as a chaperone (Glaser et al. 2012; Tripp et al. 2012). Therefore, in the present study, we investigated whether the POTRA domains of Toc75 and Tic22 form a functional complex in the chloroplast IMS, providing essential chaperone activity for the protein import process. Our results demonstrate that the POTRA domains of Toc75 and Tic22‐III exhibit functional interaction and provide the necessary chaperone activity for the import process in the IMS of chloroplasts.

First, we demonstrated that Arabidopsis Tic22‐III possesses chaperone activity, similar to Tic22 in cyanobacteria and apicoplasts (Glaser et al. 2012; Tripp et al. 2012). Using insulin as a model substrate, we demonstrated that Tic22‐III effectively prevents insulin mis‐aggregation, indicating its chaperone‐like activity (Figure 1). This activity of Tic22‐III to maintain precursor proteins in an import‐competent form, combined with the known chaperone function of Toc75 POTRA domains suggests a cooperative role in maintaining precursor proteins in an import‐ competent state within the chloroplast IMS. While Tic22‐IV is insoluble in *E. coli* in our assay conditions, consistent with previous observations (Rudolf et al. 2013), it has conserved amino acids in the non‐polar groove region similar to that of *Plasmodium falciparum* Tic22, which has been shown to be crucial for chaperone activity (Glaser et al. 2012).

Second, the *tic22‐III/TOC75∆P1* double mutant exhibited reduced chlorophyll content, compared to the respective single mutants. The genetic interaction observed here is consistent with the previously reported physical interaction between Tic22‐III and POTRA domains of Toc75 (Paila et al. 2016). It is still not clear which POTRA domain interacts with Tic22‐III physically in vivo. Whether Tic22‐III directly interacts with the incoming substrate is also not clear. However, the chlorotic phenotype of *tic22‐III/TOC75∆P1* plants as compared to their parents and the physical interaction between Tic22‐III and POTRA domains suggest that the combined chaperone activity provided by these two components is important for efficient protein import into the chloroplasts. Similarly, in cyanobacteria, *Anabaena* Tic22 has been shown to interact with Omp85, indicating a concerted genetic and physical function of both proteins (Brouwer et al. 2019). Although Tic22‐IV complements the Anabaena Tic22 functionally, the *tic22‐IV* mutant plants do not exhibit any defective phenotype as a single mutant or in combination with *tic22‐IV/TOC75∆P1* plants, which suggests that the role of Tic22‐IV has diverged in higher plants.

The reduced chlorophyll content in *tic22‐III/TOC75∆P1* plants can be attributed to the compromised functionality of chloroplast proteins resulting from improper import and folding (Figure 2). The chloroplasts isolated from *tic22‐III/TOC75∆P1* plants exhibited significantly reduced protein import capacity for both photosynthetic and non‐photosynthetic substrates (Figures 3 and 4). The reduced accumulation of LHCs, the essential thylakoid membrane proteins that bind chlorophylls and carotenoids, along with a reduced rate of protein import in *tic22‐III/TOC75∆P1*, plants underscores the combined chaperone activity of Tic22‐III and Toc75 in maintaining preproteins in an import‐competent form during their transit through the IMS.

Recently, cryo‐EM structures of TOC‐TIC supercomplexes have been reported from Chlamydomonas (Jin et al. 2022; Liu et al. 2023). However, the Tic22 protein is absent in the intermembrane space of the TOC‐TIC supercomplex when captured at stable preprotein conformations or where the protein import across both membranes is mechanistically coupled. Another attempt to purify the TOC‐TIC super complex from *Pisum sativum* during active protein import at moderate resolution could not identify any TOC complex and IMS components, suggesting that while in Chlamydomonas such complexes are stable, they are more dynamic in nature in land plants (Liang, Jin, et al. 2024). However, no Tic22 protein has been shown to be part of the TIC complex in the cryo‐EM structure from land plants. This shows that there are differences in the organization of the TOC‐TIC complexes in Chlamydomonas and land plants. Altogether, it may be possible that Tic22 remains as a soluble protein within the intermembrane space compartment and associates with the POTRA domain of the TOC complex only during active protein import. Interestingly, the cryo‐EM structure of land plants has revealed a new component in the IMS, AtTam37, which is a DnaJ‐like protein that could potentially have chaperone‐like activity. Further studies are required to fully elucidate the functions and interactions of all components within the chloroplast IMS during protein import.

In conclusion, our study provides evidence for the functional significance of Tic22‐III, its interaction with Toc75 in chloroplast protein import, and its role in maintaining chloroplast functionality. This work contributes to the broader understanding of chloroplast biogenesis and protein import, providing a foundation for further investigations into the intricate molecular mechanisms involved in these processes.

## Author Contributions

R.S: investigation, data analysis, manuscript writing. DJS: conceptualization, funding acquisition.

## Funding

This work was supported by NIH grant number 2RO 1‐GM061893 to D.J.S.

## Conflicts of Interest

The authors declare no conflicts of interest.

## Peer Review

The peer review history for this article is available in the Supporting Information for this article.

## Supporting information


**Data S1:** Peer review.

## Data Availability

All relevant data are within the paper. The material generated in the project can be provided upon request.
