# Supplementary material for: Functional Interaction Between Toc75 POTRA1 Domain and Tic22‐III in the Intermembrane Space During Chloroplast Protein Import
Source: Plant Direct. 2026 Feb 12;10(2):e70138. doi: 10.1002/pld3.70138 (PMC12895209; doi:10.1002/pld3.70138)
Supplement: Supplementary file 1 — Data S1: Peer review. [file PLD3-10-e70138-s001.docx]

Functional interaction between Toc75 POTRA1 domain and Tic22-III in the intermembrane space during chloroplast protein import

Rajneesh Singhal; Danny J. Schnell

Decisions Letter Round 1:

Dear Dr. Schnell:

Thank you for submitting to Plant Direct. All required reviews have been returned and we have now finished our evaluation of your manuscript. In light of the reviewers' and editor's comments, further revisions are needed before the paper can be accepted for publication in Plant Direct.

Please view the editors' and reviewers' comments below and use their suggestions as a guide while you work on your revision.

When uploading the revised version of this article, please be sure to include the following:

-A word document that contains your response to the reviewers. You should respond to each reviewer comment and note the changes made to the manuscript. If you do not agree with a reviewer's comment and choose not to make a suggested revision, please explain why. Please try to provide as complete an answer as possible to each reviewer's criticisms.

-A tracked changes document with each change highlighted

- A clean version of the latest version of the manuscript

To upload your revision, please click the link below.

https://plantdirect.msubmit.net/cgi-bin/main.plex?el=A1Lr6pc2A2kwM5I5A9ftd6NVQ07mO5EWLig4ftc5lgZ

In order to provide as timely a service as possible, we ask that your revision is resubmitted within three months after receipt of this request. If an extension is needed, please send a request, along with a brief explanation, to the editorial office at plantdirect@wiley.com .

Please note that, in addition to publishing reviewer comments, the author's responses to review comments will also be published alongside the final version of the paper. If you would not like the author's responses to be published, please contact the editorial office at plantdirect@wiley.com .

Thank you very much for giving us an opportunity to review your work. I look forward to receiving the next version.

Sincerely,

Yan Lu

Gustavo MacIntosh

Editor, Plant Direct

---------------------------------------------------------------------------- Editor comments:

---------------------------------------------------------------------------- Reviewer comments:

Reviewer #1:

In this manuscript, the authors have demonstrated that expression of POTRA1 domain deleted TOC75 (TOC75ΔP1) in the tic22-III mutant background resulted in a more severe phenotype than the individual mutants in terms of protein import and chlorophyll levels. These observations are novel and should be sufficient for publication in Plant Direct. I strongly suggest removing data and conclusion on protein levels and stoichiometry of other translocon components due to low data quality and incorrect techniques used (see below for details).

Major concerns:

1.Line 35: "In vitro import experiments suggest that TOC75ΔP1/tic22-III plants are compromised in protein import. Our western blot analysis and protein import studies further demonstrate that the chaperone activity provided by the Toc75/Tic22 complex is essential for importing light harvesting chlorophyll (LHC) proteins into the chloroplast stroma."

Please change the first sentence to "In vitro import experiments suggest that TOC75ΔP1/tic22-III plants are compromised in importing stromal and thylakoid membrane proteins", and then remove the second sentence. No data in this manuscript demonstrated that it is the "chaperone activity" of the two proteins that are important. There is no data for complex formation and TOC75 POTRA1 and Tic22-III are not "essential".

2.Line39: "...Toc75 POTRAs and Tic22-III act together to provide chaperone...

Please change to "......TOC75 POTRAs and Tic22-III both provide chaperone.....". There are no data showing direct interaction; the additive phenotype only suggests that they have similar functions.

3.Line 61: "...... complex consists of Tic110, Tic40, and Tic20 as core components where Tic110 and Tic40 function as scaffolds for docking the stromal chaperones."

Remove "Tic40", which is not a scaffold.

4.The statistical analysis methods described in the Materials and Methods section are inconsistent with those shown in the figure legends. Because two methods were used-Student's t-test and Duncan's test-please state clearly, for each figure, which statistical method was applied.

5.Figure 1: It would have been more convincing if the authors could include a negative control of a soluble protein that does not have chaperone activity -for example the POTRA1 domain of Toc75 as in their previous publication (O'Neil et al. 2017). If this is difficult, please add "in similar assays, purified Toc75 POTRA1 recombinant protein showed no activity (O'Neil et al. 2017)".

6.Line 242: In 7-day-old seedlings, the statistical data in Fig. 2B do not show a lower chlorophyll content in tic22-III/TOC75ΔP1 compared to tic22-III; however, in Figure S1, in 13-day old seedlings, a lower chlorophyll content in tic22-III/TOC75ΔP1 was observed. The authors should provide some explanation for this developmental change or inconsistency.

7.Line 244: "Altogether, these results suggest a functional additive interaction between the Tic22-III and POTRA2-3 domain of Toc75, while there is no ... between Tic22-IV and the POTRA2-3....."

Correct POTRA2-3 to POTRA1.

8.Line 248: ".... the tic22-III/TOC75ΔP1 plants remain pale and developmentally delayed in flowering and setting siliques compared to their parents (Fig. 2C)."

The plant picture shown in Figure 2C offers no data on "developmentally delayed in flowering and setting siliques compared to their parents". If fact, if anything, the double mutant seems to flower earlier than the TOC75ΔP1 plants. Please remove all conclusions related to developmental timing.

9.Line 264: "The accumulation levels were normalized to Tic110, a component of the inner envelope membrane, as Tic110 levels were not affected in either tic22-III, TOC75ΔP1, or in the tic22-III/TOC75ΔP1 plants (Fig. 3A, B)."

The logic is flawed here. How Tic110 level would be affected in the tic22-III/TOC75ΔP1 plants should not be known until the result of this experiment. Furthermore, if the level of Tic110 in different genotypes is used as normalization standard, meaning that the relative abundance of Tic110 in different genotype to the wild type is always set as 1 for every experiment, then why are there standard errors for the Tic110 data in different genotypes?

10.Line 272: "....levels of Toc33, another receptor component at the outer envelope membrane, were increased by 2-fold in TOC75ΔP1 and tic22-III/TOC75ΔP1 plants (Fig. 3A, B)."

The quality of the Toc33 blot is too low to make any conclusion. Also, according to Figure 3B, the increase in Toc33 in tic22-III/TOC75ΔP1 plants is not significant.

11.Line 274: "....we observed a modest but significant increase in the levels of another TIC complex component, Tic40, in TOC75ΔP1 and tic22-III/TOC75ΔP1 plants.

According to Figure 3B, there is no significant increase in Tic40 in the tic22-III/TOC75ΔP1 plants. I strongly suggest Figure 3A and 3B to be removed. There is no clear conclusion and the data quality is low.

12.Figs. 3 and 4: The statement "In this case, we also observed a significantly reduced import capacity in tic22-III/TOC75ΔP1 compared to either tic22-III or TOC75ΔP1 plants" aims to emphasize a further reduction. Therefore, the statistical test should be used to compare the tic22-III/TOC75ΔP1 vs. the tic22-III plants and the tic22-III/TOC75ΔP1 vs. TOC75ΔP1 plants, not to compare tic22-III/TOC75ΔP1 vs. the wild-type plants. The same revision should be made to Figure 4B, 4D and 4F.

13.Figure 4C and 4E: the figure legend indicated that "Coomassie stained samples indicate equal loading". However, in all 4 experiments (import of LhcA2 and B1, with or without thermolysin), more proteins are loaded in the TOC75ΔP1 sample than in the tic22-III/TOC75ΔP1 sample. This means the reduction observed in tic22-III/TOC75ΔP1 chloroplasts could simply be an artifact caused by reduced protein loading. Are the quantifications shown in Figure 4D and F corrected for protein loading? If not, the data should be recalculated.

14.Line 348: "....the 70 kDa complex (of Toc33) became more apparent in Toc75ΔP1 and tic22-III/TOC75ΔP1 plants (Fig. 5)."

This conclusion is invalid. BN-PAGE western blots are extremely difficult to quantify. Most important of all, immunoblot signals cannot be compared across blots.

15.Line 379: ".....the tic22-III/TOC75ΔP1 double mutant exhibited a more severe phenotypes, including reduced chlorophyll content and growth retardation,...."

Remove "growth retardation" as no supporting data are provided.

16.Lines 411 and 333

Remove the word "stoichiometry". Stoichiometry cannot be determined by BN-PAGE, nor by western without purified protein controls to show the exact amount of each protein.

17.Line 421: "However, Tic22 protein is not present in the intermembrane space of the TOC TIC supercomplex which has been shown to be stable at the pre-protein confirmations and the protein import across membranes is mechanistically coupled"

Please rephrase this sentence for clarity; the current version is difficult to understand.

18.It is recommended to remove Figure 6, as it is not discussed in the main text, the stoichiometry of Toc34 is incorrect, recent findings indicate that Tic214 inserts into the lumen of the Toc159-Toc75 hybrid barrel and it is never shown that Tic22IV is associated with the inner membrane.

19.The paper title is suggested to be revised to:

"Functional interaction between Toc75 POTRA1 domain and Tic22-III in the intermembrane space during chloroplast protein import."

Minor suggestions:

1. Add the method of chlorophyll content measurement in the Materials and Methods section

2. Line 293: "in tic22-III/TOC75ΔP1 plants compared to TOC75ΔP1 plants (1.68 vs. 2.4 units/min)."

Since the term units/min is undefined, please delete "(1.68 vs. 2.4 units/min)".

3. Line 56: The TOC complex includes GTPase-activity-possessing receptor components like Toc34 and Toc159"

Remove "like"

4. Line 70: ".... chaperones like Heat-shock protein 70 (Hsp70) and HSP90"In "Heat-shock" the H should be in lower case and there should not be a hyphen. Also please be consistent about whether it should be Hsp or HSP.

5. Line 71: "...At the stromal interface, ..."

It is not clear what "interface" means here. Better to just say "In the stroma,"

6. Line 90: Toc75, in Arabidopsis have three POTRA domains.

Remove "in Arabidopsis"

7. Line 250: Since we observed a functional interaction between Tic-III and Toc75ΔP1,....

It should be Tic22-III.

8. References:

Fix all errors, duplications, and incomplete entries in the reference list.

Ensure every in-text citation corresponds to a complete reference entry.

For example,

Line 65: specify whether it is Liang et al., 2024a or 2024b.

Line 73: the author "Lübeck" cannot be found in the reference list.

Remove duplicated/incomplete references such as "Ling Q, Jarvis P, Ling Q, Jarvis P. 2015," which appears twice within a single entry.

9. Legend of Figure 2B: change "Duncan T-test" to "Duncan's test"

Response Letter Round 1:

In this manuscript, the authors have demonstrated that expression of POTRA1 domain

deleted TOC75 (TOC75ΔP1) in the tic22-III mutant background resulted in a more severe

phenotype than the individual mutants in terms of protein import and chlorophyll levels.

These observations are novel and should be sufficient for publication in Plant Direct.

I sincerely thank you for your encouraging feedback on the manuscript. I greatly appreciate your

thoughtful evaluation and constructive suggestions to further improve the clarity and quality of

the manuscript.

I strongly suggest removing data and conclusion on protein levels and stoichiometry of

other translocon components due to low data quality and incorrect techniques used (see

below for details).

Thank you for your comment and suggestion. I have removed the western blot figure from

Figure3 and the BN-Page from figure 5 and the associated text with these figures as they are of

low quality.

Major concerns:

1.Line 35: "In vitro import experiments suggest that TOC75ΔP1/tic22-III plants are

compromised in protein import. Our western blot analysis and protein import studies

further demonstrate that the chaperone activity provided by the Toc75/Tic22 complex is

essential for importing light harvesting chlorophyll (LHC) proteins into the chloroplast

stroma."

Please change the first sentence to "In vitro import experiments suggest that

TOC75ΔP1/tic22-III plants are compromised in importing stromal and thylakoid

membrane proteins", and then remove the second sentence. No data in this manuscript

demonstrated that it is the "chaperone activity" of the two proteins that are important.

There is no data for complex formation and TOC75 POTRA1 and Tic22-III are not

"essential".

I thank you for your comment. I have performed the edits as suggested in the abstract on lines

35-36.

2.Line39: "...Toc75 POTRAs and Tic22-III act together to provide chaperone...

Please change to "......TOC75 POTRAs and Tic22-III both provide chaperone.....". There

are no data showing direct interaction; the additive phenotype only suggests that they have

similar functions.

I thank you for your comment. I have performed the edits as suggested in the abstract on line 37

and then again in Introduction on line 100.

3.Line 61: "...... complex consists of Tic110, Tic40, and Tic20 as core components where

Tic110 and Tic40 function as scaffolds for docking the stromal chaperones."

Remove "Tic40", which is not a scaffold.

I thank you for your comment. I have performed the edits as suggested on line 59.

4.The statistical analysis methods described in the Materials and Methods section are

inconsistent with those shown in the figure legends. Because two methods were usedStudent's t-test and Duncan's test-please state clearly, for each figure, which statistical

method was applied.

I thank you for your comments and suggestions. To make it consistent among all the figures I

have redone the statistical analysis using Tukey’s HSD emmeans package in R. I have added the

methodology in materials and method section on lines 184-190, and below each figure legend.

5.Figure 1: It would have been more convincing if the authors could include a negative

control of a soluble protein that does not have chaperone activity -for example the

POTRA1 domain of Toc75 as in their previous publication (O'Neil et al. 2017). If this is

difficult, please add "in similar assays, purified Toc75 POTRA1 recombinant protein

showed no activity (O'Neil et al. 2017)".

I thank you for your comment. Since it was not possible for me to do the experiment with a

negative control, I added the sentence suggested by you at lines 208-209.

6.Line 242: In 7-day-old seedlings, the statistical data in Fig. 2B do not show a lower

chlorophyll content in tic22-III/TOC75ΔP1 compared to tic22-III; however, in Figure S1, in

13-day old seedlings, a lower chlorophyll content in tic22-III/TOC75ΔP1 was observed. The

authors should provide some explanation for this developmental change or inconsistency.

I thank you for your thoughtful insight at this observation. Yes, the Fig 2B do not show a

statistically significant chlorophyll content in tic22-III/TOC75ΔP1 as compared to tic22-III. the

figure shows that tic22-III has a significantly reduced chlorophyll content as compared to WT

and TOC75ΔP1, and there is no significant difference between WT and TOC75ΔP1. However, at

13 d, tic22-III recovers from its chlorophyll defect but the tic22-III/TOC75ΔP1 have statistically

significant lower chlorophyll content as compared to all the other genotypes. While the plants

recover from tic22-III deletion as they grow, the expression of TOC75P1 in tic22-III mutant

background is still chlorotic suggesting that the POTRA1 domain of TOC75 might be important

for providing stability to the incoming precursor protein in concert with TIC22-III protein. To

highlight it in better way, I have moved the supplemental figure S1 to the main figure as part

Figure 2C and 2D. I have added the following text in the results section in lines 230-239.

“tic22-IV mutants, and TOC75ΔP1 seedlings are indistinguishable from WT, while tic22-III

mutants show a pale phenotype and a deficiency in chlorophyll (Fig. 2A, B). However, while

tic22-IV/TOC75ΔP1 seedlings are phenotypically similar to TOC75ΔP1, the tic22-III/TOC75ΔP1

plants have enhanced chlorotic phenotype similar to tic22-III. Interestingly, while tic22-III

seedlings recovered from chlorophyll defect by day 13, the tic22-III/TOC75ΔP1 seedlings fail to

recover and retain significantly lower chlorophyll content than all other genotypes (Fig 2C, D).

The triple mutant tic22-III/tic22-IV/TOC75ΔP1 plants are indistinguishable from tic22-

III/TOC75ΔP1 plants. The failure of tic22-III/TOC75ΔP1 seedlings to recover from chlorophyll

defect implies a specific functional interaction between Tic22-III and the POTRA1 domain of

Toc75 (Fig. 2D).”

I hope that this address your concern regarding this figure.

7.Line 244: "Altogether, these results suggest a functional additive interaction between the

Tic22-III and POTRA2-3 domain of Toc75, while there is no ... between Tic22-IV and the

POTRA2-3....."

Correct POTRA2-3 to POTRA1.

I have modified the sentence to reflect the suggestion on line 237-239: “The failure of tic22-

III/TOC75ΔP1 seedlings to recover from chlorophyll defect implies a specific functional

interaction between Tic22-III and the POTRA1 domain of Toc75 (Fig. 2D).”

8.Line 248: ".... the tic22-III/TOC75ΔP1 plants remain pale and developmentally delayed

in flowering and setting siliques compared to their parents (Fig. 2C)."

The plant picture shown in Figure 2C offers no data on "developmentally delayed in

flowering and setting siliques compared to their parents". If fact, if anything, the double

mutant seems to flower earlier than the TOC75ΔP1 plants. Please remove all conclusions

related to developmental timing.

I thank you for your suggestion. I agree with the reviewer that the figure does not offer any

conclusion regarding developmental delay and does not convey much information, I have

removed the figure and associated conclusion from the paper.

9.Line 264: "The accumulation levels were normalized to Tic110, a component of the inner

envelope membrane, as Tic110 levels were not affected in either tic22-III, TOC75ΔP1, or in

the tic22-III/TOC75ΔP1 plants (Fig. 3A, B)."

The logic is flawed here. How Tic110 level would be affected in the tic22-III/TOC75ΔP1

plants should not be known until the result of this experiment. Furthermore, if the level of

Tic110 in different genotypes is used as normalization standard, meaning that the relative

abundance of Tic110 in different genotype to the wild type is always set as 1 for every

experiment, then why are there standard errors for the Tic110 data in different genotypes?

I thank you for your insight in this figure. I apologize for the error here. The reason we used

Tic110 as a control for the western in this figure was based on the observation of previous paper

(Paila et al., 2016) where Tic110 level were not affected in various TOC75 POTRA deletions.

And not on the sentence mentioned above. You are correct to point out that since I have

normalized it to Tic110 the error bars in the Tic110 are confusing and should not be there. But

since you have later suggested to remove the figure, I have removed it from the figure panel and

all the associated results and conclusions regarding the western data.

10.Line 272: "....levels of Toc33, another receptor component at the outer envelope

membrane, were increased by 2-fold in TOC75ΔP1 and tic22-III/TOC75ΔP1 plants (Fig.

3A, B)."

The quality of the Toc33 blot is too low to make any conclusion. Also, according to Figure

3B, the increase in Toc33 in tic22-III/TOC75ΔP1 plants is not significant.

I agree with you and as suggested later, I have removed the figure.

11.Line 274: "....we observed a modest but significant increase in the levels of another TIC

complex component, Tic40, in TOC75ΔP1 and tic22-III/TOC75ΔP1 plants.

According to Figure 3B, there is no significant increase in Tic40 in the tic22-III/TOC75ΔP1

plants. I strongly suggest Figure 3A and 3B to be removed. There is no clear conclusion and

the data quality is low.

I thank you for your suggestion and have removed the figure. As such Figure 3 now has import

data only. I have removed the paragraph discussing western data from results and discussion

sections.

12.Figs. 3 and 4: The statement "In this case, we also observed a significantly reduced

import capacity in tic22-III/TOC75ΔP1 compared to either tic22-III or TOC75ΔP1 plants"

aims to emphasize a further reduction. Therefore, the statistical test should be used to

compare the tic22-III/TOC75ΔP1 vs. the tic22-III plants and the tic22-III/TOC75ΔP1 vs.

TOC75ΔP1 plants, not to compare tic22-III/TOC75ΔP1 vs. the wild-type plants. The same

revision should be made to Figure 4B, 4D and 4F.

I thank you for your constructive suggestions. I have redone the statistical test using Tukey’s

HSD for multiple comparison between different genotypes for all the figures and used compact

letter display to show the differences between genotypes on the figure. Now the comparison is

more obvious among all the genotypes. I have also rewritten text to indicate the comparison

among genotypes.

For figure 3A,B (pSSU import): no changes in the result section.

For Figure 3 C, D (import of E1a) the corresponding change is made on lines 254-257.

For Figure 4A,B (western): no changes in the result section.

For Figure 4 D, F: text changes on lines 284-291 in result section.

13.Figure 4C and 4E: the figure legend indicated that "Coomassie stained samples indicate

equal loading". However, in all 4 experiments (import of LhcA2 and B1, with or without

thermolysin), more proteins are loaded in the TOC75ΔP1 sample than in the tic22-

III/TOC75ΔP1 sample. This means the reduction observed in tic22-III/TOC75ΔP1

chloroplasts could simply be an artifact caused by reduced protein loading. Are the

quantifications shown in Figure 4D and F corrected for protein loading? If not, the data

should be recalculated.

I thank you for your keen observations and suggestions here. I have recalculated the data as

suggested by normalizing to the rubisco band shown in the Coomassie stained gel and have

redrawn the graph. I have modified the text in the figure legend to reflect this normalization step.

I have also rewritten the result section as follows on lines 284-291:

“As shown in Fig. 4 (C-F), there was a significant decrease in the import rate of both

LhcA2 and LhcB1 in tic22-III/TOC75ΔP1 plants compared to WT. However, the extent of the

reduction compared to tic22-III or TOC75ΔP1 plants differed between two proteins. For LhcA2

the rate of import in tic22-III/TOC75ΔP1 plants was comparable to TOC75ΔP1 mutant but lower

than tic22-III. In contrast, LhcB1 import in tic22-III/TOC75ΔP1 plants was significantly reduced

compared to both tic22-III or TOC75ΔP1 single mutant plants. This defect in import suggests

that both Tic22-III and POTRA1 domain of Toc75 protein cooperate in the IMS for the import of

hydrophobic proteins.”

14.Line 348: "....the 70 kDa complex (of Toc33) became more apparent in Toc75ΔP1 and

tic22-III/TOC75ΔP1 plants (Fig. 5)."

This conclusion is invalid. BN-PAGE western blots are extremely difficult to quantify. Most

important of all, immunoblot signals cannot be compared across blots.

I thank you for your suggestion. Since the blot of low quality, I have removed it from the paper.

15.Line 379: ".....the tic22-III/TOC75ΔP1 double mutant exhibited a more severe

phenotypes, including reduced chlorophyll content and growth retardation,...."

Remove "growth retardation" as no supporting data are provided.

I have performed the edits as suggested in the discussion section on lines 318-319.

16.Lines 411 and 333

Remove the word "stoichiometry". Stoichiometry cannot be determined by BN-PAGE, nor

by western without purified protein controls to show the exact amount of each protein.

I thank you for your suggestion. I have removed the section on BN-Page and the related text

from result and discussion section.

17.Line 421: "However, Tic22 protein is not present in the intermembrane space of the

TOC TIC supercomplex which has been shown to be stable at the pre-protein

confirmations and the protein import across membranes is mechanistically coupled"

I thank you for your comment. I have repharased the sentence to make it clearer on lines 341-

343 as follows: “ However, Tic22 protein is absent in the intermembrane space of the TOC-TIC

supercomplex when captured at stable preprotein conformations or where the protein import

across both membranes is mechanistically coupled.”

18.It is recommended to remove Figure 6, as it is not discussed in the main text, the

stoichiometry of Toc34 is incorrect, recent findings indicate that Tic214 inserts into the

lumen of the Toc159-Toc75 hybrid barrel and it is never shown that Tic22IV is associated

with the inner membrane.

I thank you for your comment. I have removed the figure as suggested.

19.The paper title is suggested to be revised to:

"Functional interaction between Toc75 POTRA1 domain and Tic22-III in the

intermembrane space during chloroplast protein import."

I have revised the title of the paper as suggested.

Minor suggestions:

1. Add the method of chlorophyll content measurement in the Materials and Methods

section

I apologize for this negligence. I have added the method on chlorophyll content measurement in

materials and methods section on line 147-154.

2. Line 293: "in tic22-III/TOC75ΔP1 plants compared to TOC75ΔP1 plants (1.68 vs. 2.4

units/min)."

Since the term units/min is undefined, please delete "(1.68 vs. 2.4 units/min)".

I have deleted it.

3. Line 56: The TOC complex includes GTPase-activity-possessing receptor components

like Toc34 and Toc159"

Remove "like"

removed as suggested.

4. Line 70: ".... chaperones like Heat-shock protein 70 (Hsp70) and HSP90"

In "Heat-shock" the H should be in lower case and there should not be a hyphen. Also

please be consistent about whether it should be Hsp or HSP.

Performed edits as suggested.

5. Line 71: "...At the stromal interface, ..."

It is not clear what "interface" means here. Better to just say "In the stroma,"

performed edits as suggested.

6. Line 90: Toc75, in Arabidopsis have three POTRA domains.

Remove "in Arabidopsis"

removed.

7. Line 250: Since we observed a functional interaction between Tic-III and Toc75ΔP1,....

It should be Tic22-III.

The sentence have been deleted.

8. References:

Fix all errors, duplications, and incomplete entries in the reference list.

Ensure every in-text citation corresponds to a complete reference entry.

For example,

Line 65: specify whether it is Liang et al., 2024a or 2024b.

Line 73: the author "Lübeck" cannot be found in the reference list.

Remove duplicated/incomplete references such as "Ling Q, Jarvis P, Ling Q, Jarvis P.

2015," which appears twice within a single entry.

I thank you for comments. I have checked every reference and now they are in order.

9. Legend of Figure 2B: change "Duncan T-test" to "Duncan's test"

I have redone the calculations using Tukey’s HSD and have added that information in all the

figure panels.
